# Analysis on the polymorphisms of site RS4977574, and RS1333045 in region 9p21 and the susceptibility of coronary heart disease in Chinese population

**DOI:** 10.1186/s12881-020-0965-x

**Published:** 2020-02-17

**Authors:** Lei Hua, Jin-Xia Yuan, Shu He, Chen-Hui Zhao, Qiao-Wei Jia, Jing Zhang, Feng-Hui An, Zhao-Hong Chen, Li-Hua Li, Lian-Sheng Wang, Wen-Zhu Ma, Guang-Xu Xu, En-Zhi Jia

**Affiliations:** 1 Department of Cardiovascular Medicine, The First Affiliated Hospital of Nanjing Medical University, Guangzhou Road 300, Nanjing, Jiangsu Province, 210029 China; 2 Department of Cardiovascular Medicine, The Friendship Hospital of Ili Kazakh Autonomous Prefecture, Yining, China; 3 Department of Rehabilitation Medicine, The First Affiliated Hospital of Nanjing Medical University, Nanjing, China

**Keywords:** Coronary heart disease, SNP, Chromosome 9p21

## Abstract

**Background:**

Rs4977574 (A > G) and Rs1333045 (C > T) are both single nucleotide polymorphisms (SNPs) related with coronary artery disease, locating on chromosome 9p21.3. The study aimed to identify the correlation between rs4977574 and rs1333045 polymorphism genotypes and coronary heart disease (CHD) in a Chinese population.

**Methods:**

Blood samples were collected from 855 subjects. A case-control study was used in this experiment, and 598 cases in the CHD group and 257 subjects in the control group were enrolled. Genotyping was identified by the Agena MassARRAY system. Statistical analysis was conducted by SPSS (Ver 16.0) and plink (Ver. 1.07, Shaun Purcell). Haplotype analysis was performed using Haploview software.

**Results:**

Association analysis by plink indicated a significant difference in the allele distribution for single nucleotide polymorphisms between cases and controls (rs4977574 *P* = 0.003, rs1333045 *P* = 0.035). Fisher’s exact test by plink proved that allele G may be associated with a higher risk of CHD (*P* = 0.003, odds ratio (*OR*) *= 1.371*) and the T allele was likely to reduce the risk of coronary events (*P* = 0.035, *OR = 0.798*). The serum levels of apolipoprotein A (ApoA) were higher in subjects with the AG + AA genotype of rs4977574 compared to those with the GG genotype (*P* = 0.028). In the dominant model of rs1333045, the levels of ApoA were higher and LDL levels were lower in the TC + TT genotype than in the CC genotype.

**Conclusions:**

The present study examined the association between the 9p21 chromosome rs4977574 and rs1333045 polymorphism genotypes and CHD in a population of Chinese patients. The G allele of rs4977574 and the C allele of rs1333045 are the susceptibility sites of CHD.

## Background

Coronary heart disease (CHD) is coronary atherosclerotic heart disease. CHD often occurs due to coronary atherosclerosis, and the rupture of the atherosclerotic plaque contributes to thrombosis, resulting in myocardial infarction, myocardial remodeling, heart failure, and even death. CHD is the top cause of human death in the United States [1] and Asian countries such as China [2] and Japan [3]. CHD has been a hot topic in medical research because of its high incidence and high mortality. New research shows that CHD is a complex disease caused by a combination of genetic and environmental factors [4]. In addition to traditional risk factors such as age, smoking, high blood pressure and abnormal blood lipids, the combination of disease susceptibility genes and environmental factors affects the development of CHD. Identifying the susceptibility gene for coronary heart disease can help in early diagnosis and treatment. However, the exact mechanism of the disease is still unclear. In recent years, genetic predisposition has been thought to be closely associated with CHD and has been widely studied [5, 6].

Based on genome-wide association studies (GWAS), one of the important loci associated with CHD is the 9p21 locus, which has been introduced by the deCODE database. CDKN2B-AS1 of antisense noncoding RNA in the INK4 locus (ANIRL) is an antisense long noncoding RNA (lncRNA) mapped on the 9p21 locus [7] consisting of 19 exons, spanning 126.3 kb in the genome. The gene contributes to epigenetic inhibition of transcription by regulating Polycomb proteins [8]. According to the published literature, this gene is involved in developing cardiovascular diseases, diabetes, Alzheimer’s disease, and several types of cancer [9–11]. Regulation of cardiac CDKN2B-AS1 expression has been found to play a pivotal role in the development of CHD by altering the dynamics of vascular cell proliferation [12]. Rs4977574 is a nonprotein coding SNP (A > G) that is located in proximity to the cyclin-dependent kinase inhibitor 2A and B genes on chromosome 9p21.3. Rs4977574 has been recently found to be associated with the early onset of CHD, which is differentially expressed in a variety of tissues, such as vascular endothelial cells and smooth coronary muscle cells [13–16], and rs4977574 is characterized by a guanine nucleotide (G) instead of an adenine nucleotide (A) [17]. rs1333045 (C > T) is also an artery disease susceptibility SNP located in a conserved region in CDKN2B-AS1 that has been shown to have enhancer activity in a reporter gene experiment [13]. However, the underlying mechanisms in the regulation of rs4977574 and rs1333045 to CHD are unknown, and there is a lack of genetic evidence describing the pathogenesis in greater detail. Giving its limitations, coronary arteriography remains the gold standard for documenting the extent and severity of CHD.

Therefore, to provide a more detailed description of the correlation between Rs4977574 (A > G) and Rs1333045 (C > T) and CHD in a Chinese population, we conduct a case and control study by analysing the datasets collected from 855 subjects. It demonstrates that the G allele of rs4977574 and the C allele of rs1333045 are the susceptibility sites of CHD, laying a foundation for further study about the mechanisms in the regulation of rs4977574 and rs1333045 to CHD.

## Methods

### Study subjects

All selected subjects signed informed consent, and the study was approved by the ethics committee of the First Affiliated Hospital of Nanjing Medical University and the Friendship Hospital of Ili Kazakh Autonomous Prefecture in China. There were 855 cases, including 629 males and 226 females, with an average age of 60 years. Patients with spastic angina pectoris, infections within the previous 2 weeks, heart failure, adrenal dysfunction, and thyroid dysfunction were excluded from the study.

In the CHD group, we selected a total of 598 patients (465 males and 133 females) aged 61 (53–70) years who were clinically diagnosed with CHD in the Friendship Hospital of Ili Kazakh Autonomous Prefecture in China from March 1, 2010 to April 31, 2015. The patients were confirmed by coronary angiography, which was performed by at least two experienced doctors simultaneously. In addition, 257 subjects (164 males and 93 females) aged 58 (49.5–66) years with negative angiography were selected as the control group. Coronary arteries were cannulated using either the Judkins technique [18] or a radial artery approach with 6F catheters. CHD subjects were defined as at least one major epicardial vessel with > 50% stenosis; control subjects were defined as all of the major epicardial vessels with < 50% stenosis [19]. All subjects were unrelated Chinese to ensure the consistency of genetic background.

### Laboratory investigation

In this study, four milliliters of peripheral blood was drawn from the subjects’ veins after 12 h of fasting to perform biochemical assays on the second day of hospitalization. Each assay index was the reported clinical parameter associated with coronary heart disease [20–26]. Total cholesterol (TC, mmol/L), triglyceride (TG, mmol/L), creatinephosphokinase isoenzyme (CKMB, U/L), fasting high-density lipoprotein cholesterol (HDL-C, mmol/L), fasting low-density lipoprotein cholesterol (LDL-C, mmol/L), apolipoprotein A (ApoA, g/L), apolipoprotein B (ApoB, g/L), glucose (Glu, mmol/L), blood urea nitrogen (Bun, mmol/L) and serum creatinine (Cre, μmol/L) were detected by the enzymatic method with an automated autoanalyzer (AU 2700 Olympus, 1st Chemical Ltd., Japan). Blood pressure was measured in the bare right arm with the participant seated, and the results were averaged over three measurements.

### Smoking and drinking

For smoking, patients were classified as either nonsmokers (never smoking) or smokers (smoking now or in the past). Patients who smoked one cigarette a day were classified as current smokers. For drinking, patients were classified as nondrinkers (never having a drink) or drinkers (drinking now or in the past). Patients who drank at least 50 g of alcohol a week were considered drinkers.

### Hypertension

Patients were defined as having hypertension if they had been previously diagnosed with hypertension and took antihypertensive drugs or if their systolic blood pressure was 140 mmHg or their diastolic blood pressure was 90 mmHg.

### Scoring of coronary angiogram

Coronary angiography results were scored according to Gensini’s scoring system. In Gensini’s scoring system [27], the degree of coronary stenosis in the range of 0–25% was scored as 1 point, stenosis in the range of 25–50% was scored as 2 points, 50–75% was scored as 4 points, 75–90% was scored as 8 points, 90–99% was scored as 16 points, and completely occluded was scored as 32 points. Every stenosed segment was divided into 0.5 to 5 according to the importance of the segment’s function. According to Gensini’s standard, each major vessel and segment were graded, and the total score was calculated to show the severity of coronary lesion.

### DNA extraction and determination of SNP genotypes

**Fig. 1:**
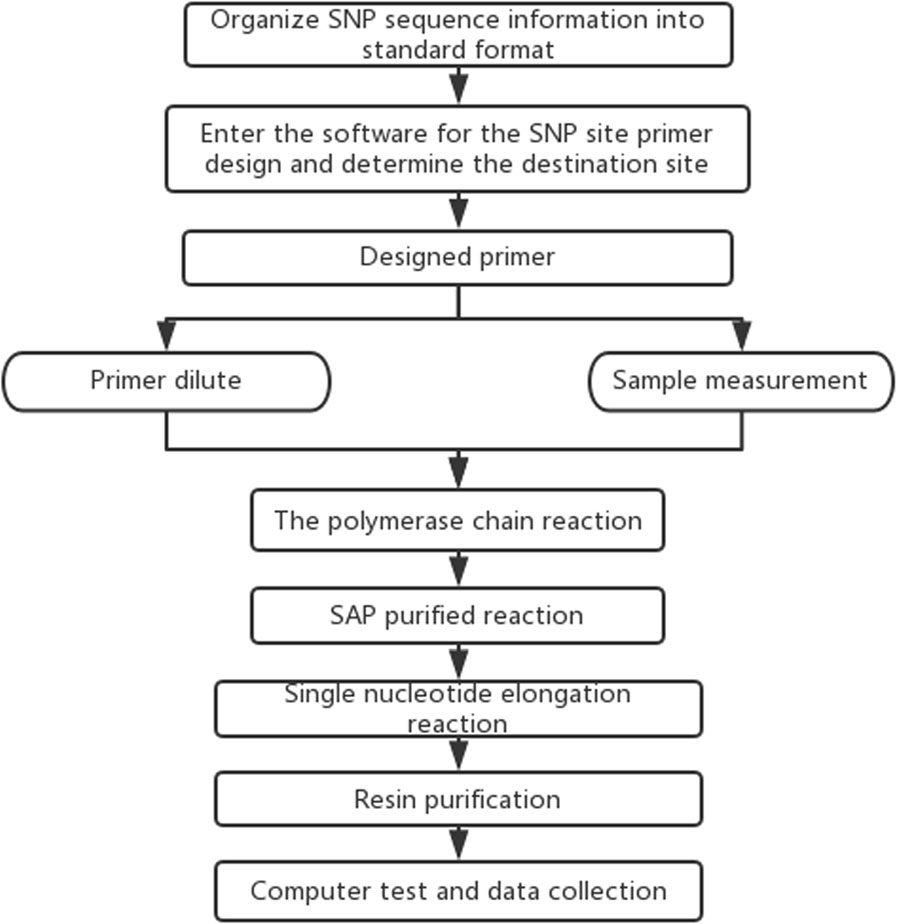
The experimental flow graph of MassARRAY SNP typing

The experimental process of MassARRAY SNP typing is shown in Fig. 1. Gene polymorphisms were identified on the Agena MassARRAY system (Agena/Sequenom Inc., San Diego, CA, USA) according to the manufacturer’s instructions. The literature and AssayDesigner3.1 software (Sequenom Inc., San Diego, CA, USA) were both used to design polymerase chain reaction (PCR) and single base extension primers. The primers were diluted for backup analysis by a professional biotech company.

The DNA in blood samples, tissues, cells and saliva was extracted using the finished product kit [Axygene Biotechnology (Hangzhou) Limited, Hangzhou City, China]. All DNA quality checks were qualified by using the NanoDrop2000 instrument (Thermo, Wilmington, DE, USA) for OD value detection and 1.25% agarose gel electrophoresis detection. All samples were transferred to a 96-well plate and stored for reserve at − 20 °C. DNA samples were amplified using standard PCR. The PCR master mix was configured in a 1.5 ml EPtube and oscillated at low speed centrifugation. Eight or 12 channel shifters were chosen, and 4 μL of PCR master mix were added to each well in 384 well plate, along with 1 μL of template DNA (20 ng/μL). The 384-well plate was carefully sealed and pressed over each well to prevent evaporation during the PCR procedure. The plate was centrifuged at 1000 RPM for 1 min. The reaction environment and procedures were set as 94 °C, 5 min; 94 °C, 20 s; 56 °C, 30 s; 72 °C, 1 min; 72 °C, 3 min. Forty-five amplification cycles were performed, and the reactants were stored at 4 °C. The PCR products were treated with shrimp alkaline phosphatase (SAP) to remove the free dNTPs in the system. Five microliters of PCR products and 2 μL of SAP mix were mixed in 384-well plates. The SAP reaction program was set to 37 °C for 20 min and heated to 85 °C for 5 min after centrifugation. The products were stored at 4 °C. Two microliters of extend mix and 7 μL of PCR + SAP reaction reagent were mixed in each well of 384-well plates. The products were transferred into 384-well spectroCHIP bioarray by MassARRAY Nanodispenser RS1000 sampler (Agena, Inc.). The gene chip was analyzed by mass spectrometry (MALDI-TOF-MS). The original data and genotypes were obtained using MassARRAY TYPER 4.0 software (Agena, Inc).

### Statistical analysis

All statistical analyses were performed with the Statistics Package for Social Sciences statistical software (Ver. 16.0, Chicago, USA) and plink (Ver. 1.07, Shaun Purcell). Quantitative variables are presented as the mean ± standard deviation, while qualitative variables are expressed as percentages. Skewed data are presented as the median (interquartile range). Smoking status, drinking status, hypertension, gender and the SNP site were examined by the chi-square test. The serum level of ApoB was examined by independent samples *t* test, and the remaining baseline characteristics were examined by the Mann-Whitney test for the demographics of the study population. To detect clinical parameters distributed in different genotypes, the serum level of ApoB was examined by one-way ANOVA, and the remaining baseline characteristics were examined by the Kruskal-Wallis test. Fisher’s exact test was used for the distribution of alleles between the two groups. Logistic regression analysis was used to assess the significance of the association between SNP rs4977574, rs1333045 and CHD. Comparison of the genotype and environmental factors between cases and controls was determined by multivariable logistic regression analysis. The odds ratio (*OR*) and 95% confidence intervals (*CI*) were also calculated. The Hardy-Weinberg law tests the equilibrium of allele frequency and genotype frequency in two groups. Pairwise linkage disequilibrium (*LD*) between sites was assessed by calculating D’ and squared correlation (*r*^*2*^) using Haploview software. *P* < 0.05 was considered statistically significant between the two groups.

The effects of the combination of gene and environment include not only the effects of both genes but also the superposition of gene and environmental effects and the multiplicative effect of gene and environmental effects. Through different models, we can distinguish the existence and size of interactions between two factors based on different models. Based on the additive model, there are several indicators for calculating interaction: (1) the interaction index (the synergy index *S*, *S*): when *S* = 1, no interaction; when *S* > 1, there is a positive interaction between the two factors; when *S* < 1, there is a negative interaction between the two factors. (2) The attribution ratio of interaction (attributable proportion of interaction *AP*): it shows how much of the total effect is attributable to two-factor interactions. (3) The attribution ratio of pure interaction: AP* indicates the proportion of interaction between two factors, which is caused by two factors of gene and environment. (4) The interaction excess relative risk degree (relative excess risk of interaction, *RERI*): this indicator indicates the difference between the combined effect of two factors and the sum of their individual effects and indicates the relationship between interaction and the effect of factors other than the two factors. If the unknown factor plays a great role, the interaction of the study becomes very minor and meaningless. Here, *RERI* is the two-factor interaction value based on the additive model.

## Results

### Hardy-Weinberg equilibrium

Hardy-Weinberg equilibrium (HWE) analysis revealed that the two study groups showed a remarkable genetic disequilibrium for both genotypes of rs4977574 and rs1333045 polymorphisms in this study, and the two groups were representative of the group.

### Characteristics of the subjects

All 855 Chinese participants were genotyped for the rs4977574 and rs1333045 SNPs and included in the study. In this study, the CHD group included 465 males and 133 females, and the control group included 164 males and 93 females. The clinical characteristics of the participants are shown in Table 1. There were significant differences in age (*P*<0.001) and gender (*P*<0.001) between the CHD and control groups; older or male subjects are presented in the CHD group. Smokers (*P* = 0.031) are more susceptible to CHD, and the incidence of hypertension (*P* = 0.008) in the CHD group was significantly increased. The serum levels of CKMB (*P* = 0.005) in the CHD group were higher than in the control group, while the levels of HDL (*P* = 0.015) were much lower. In addition, the content of Glu (*P*<0.001) and Cre (*P* = 0.003) in CHDs was significantly higher than that in the controls. These variables are likely to be associated with CHD. The levels of TC and TG revealed no strong differences between CHDs and controls, which may be related to the use of lipid-lowering drugs in hospitalized patients.

**Table 1:** Demographics of the study population

Variables	CHDs (*n* = 598)	Controls (*n* = 257)	Statistical parameter	*P* value
Age (years)	61 (53–70)	58 (49.5–66)	−3.839	< 0.001
Gender (male/ female)	465/133	164/93	17.978	< 0.001
Smoking Status (yes/no)	283/315	101/156	4.679	0.031
Drinking Status (yes/no)	96/502	34/223	1.112	0.292
Hypertension (yes/no)	324/274	114/143	6.941	0.008
CKMB (U/L)	17 (13–23)	16 (13–20)	−2.804	0.005
TC (mmol/L)	4.65 (3.88–5.52)	4.60 (3.91–5.38)	−0.837	0.403
TG (mmol/L)	1.80 (1.25–2.48)	1.71 (1.16–2.46)	− 1.139	0.255
HDL (mmol/L)	1.33 (1.10–1.62)	1.41 (1.18–1.68)	−2.426	0.015
LDL (mmol/L)	2.79 (2.19–3.48)	2.71 (2.17–3.38)	−1.325	0.185
ApoA (g/L)	1.29 (1.14–1.45)	1.30 (1.18–1.49)	−1.747	0.081
ApoB (g/L)	0.92 ± 0.23	0.90 ± 0.21	−1.234	0.218
Glu (mmol/L)	5.19 (4.65–6.16)	4.84 (4.53–5.34)	−5.447	< 0.001
Bun (mmol/L)	4.90 (4.05–5.88)	4.87 (4.05–5.80)	−0.241	0.809
Cre (μmol/L)	72 (62–81.25)	69 (60–78)	−2.937	0.003
RS4977574 (GG/AG/AA)	152/297/149	48/122/87	8.686	0.012
RS1333045 (TT/TC/CC)	138/292/168	73/127/57	4.470	0.107

Skewed data are presented as the median (interquartile range), normal data are presented as the mean ± standard deviation, and categorical data are presented as the absolute value. Smoking status, drinking status, hypertension, gender and the SNP site were examined by the chi-square test. The serum level of ApoB was examined by the independent samples *t* test, and the remaining baseline characteristics were examined by the Mann-Whitney test

### Clinical parameter distribution in different genotypes

According to Table 1, the subjects were divided into three groups according to different genotypes: GG represents the homozygote of minor alleles, AG represents the heterozygote, and AA represents the homozygote of major alleles in rs4977574. TT represents the homozygote of minor alleles, TC represents the heterozygote, and CC represents the homozygote of major alleles in rs1333045. The three groups of genotypes were compared regarding clinical parameters, and the results are summarized in Table 2. Accordingly, no significant difference was observed between the frequencies of rs4977574 and rs1333045 in the distribution of age, gender, smoking status, drinking status, hypertension, CKMB, TC, TG, HDL, LDL, ApoA, ApoB, Glu, Bun and Cre (*P* > 0.05). However, the Gensini score did differ significantly among the rs4977574 genotypes (*P* = 0.017). This indicates that the G allele was associated with the severity of CHD. The allele frequency in subjects is all consistent with HWE (*P* = 0.596 for rs4977574, *P* = 0.566 for rs1333045).

**Table 2:** Clinical parameters distributed in different genotypes

RS4977574	GG (*n* = 200)	AG (*n* = 419)	AA (*n* = 236)	*P* value
Age (years)	60 (51–68)	60 (52–69)	61 (52.25–68)	0.604
Gender (male/ female)	143/57	311/108	175/61	0.750
Smoking (yes/no)	89/111	189/230	106/130	0.990
Drinking (yes/no)	26/174	66/353	38/198	0.607
Hypertension (yes/no)	99/101	218/201	121/115	0.841
CKMB (U/L)	17 (13–22)	17 (13.40–21)	16 (12.10–21)	0.145
TC (mmol/L)	4.61 (3.93–5.48)	4.63 (3.92–5.40)	4.64 (3.75–5.61)	0.883
TG (mmol/L)	1.85 (1.26–2.41)	1.82 (1.23–2.55)	1.68 (1.13–2.43)	0.188
HDL (mmol/L)	1.32 (1.10–1.62)	1.37 (1.14–1.63)	1.41 (1.15–1.67)	0.208
LDL (mmol/L)	2.83 (2.26–3.45)	2.77 (2.15–3.40)	2.71 (2.14–3.53)	0.394
ApoA (g/L)	1.27 (1.13–1.42)	1.30 (1.16–1.46)	1.30 (1.16–1.46)	0.086
ApoB (g/L)	0.90 ± 0.21	0.93 ± 0.22	0.91 ± 0.24	0.305
Glu (mmol/L)	5.04 (4.55–5.80)	5.07 (4.63–6.00)	5.09 (4.59–6.07)	0.504
Bun (mmol/L)	4.87 (4.02–5.61)	4.87 (4.06–6.08)	4.94 (3.96–5.80)	0.381
Cre (μmol/L)	69 (61–81)	72 (62–82)	70 (62–79.75)	0.182
Gensini	20 (5–45.75)	18 (3–48)	10 (1.25–40)	0.017
HWE				0.596
RS1333045	TT (*n* = 211)	TC (*n* = 419)	CC (*n* = 225)	*P* value
Age (years)	61 (53–68)	60 (52–70)	60 (51.5–68)	0.697
Gender (male/ female)	159/52	304/115	166/59	0.751
Smoking (yes/no)	97/114	185/234	102/123	0.901
Drinking (yes/no)	38/173	63/356	29/196	0.327
Hypertension (yes/no)	106/105	218/201	114/111	0.896
CKMB (U/L)	16 (12.4–21)	17 (13–21)	17 (13–22)	0.446
TC (mmol/L)	4.60 (3.74–5.62)	4.65 (3.93–5.40)	4.62 (3.91–5.51)	0.933
TG (mmol/L)	1.71 (1.15–2.46)	1.74 (1.19–2.48)	1.90 (1.27–2.47)	0.168
HDL (mmol/L)	1.38 (1.13–1.67)	1.38 (1.14–1.63)	1.32 (1.11–1.62)	0.336
LDL (mmol/L)	2.64 (2.09–3.48)	2.75 (2.18–3.36)	2.94 (2.27–3.51)	0.087
ApoA (g/L)	1.29 (1.15–1.46)	1.30 (1.17–1.49)	1.27 (1.13–1.43)	0.129
ApoB (g/L)	0.91 ± 0.25	0.93 ± 0.22	0.90 ± 0.21	0.382
Glu (mmol/L)	5.10 (4.59–6.04)	5.05 (4.63–6.02)	5.07 (4.57–5.79)	0.493
Bun (mmol/L)	4.85 (3.91–5.74)	4.88 (4.07–6.14)	4.93 (4.04–5.78)	0.392
Cre (μmol/L)	71 (62.8–80)	62 (71–82)	69 (61–81)	0.463
Gensini	12 (2–40)	16 (3–45)	20 (4–48)	0.088
HWE				0.566

HWE: *P* value for Hardy-Weinberg equilibrium test. Skewed data are presented as the median (interquartile range), normal data are presented as the mean ± standard deviation, and categorical data are presented as the absolute value. The serum level of ApoB was examined by one-way ANOVA, and the remaining baseline characteristics were examined by Kruskal-Wallis tests

### Serological biomarkers in the dominant model and recessive model

Table 3 shows the serological biomarkers in the dominant model (rs4977574, AA vs. AG + GG; rs1333045, CC vs. TC + TT) and recessive model (rs4977574, GG vs. AA+AG; rs1333045, TT vs. TC + CC). Table 3 shows that the serum levels of ApoA in the GG genotype were significantly lower than those in the AA+AG genotype (*P* = 0.028). In the dominant model of rs1333045, the serum levels of LDL in the CC genotype were higher than those in the TC + TT genotype (*P* = 0.032). In contrast, the serum levels of ApoA in the CC genotype were significantly lower than those in the TC + TT genotype (*P* = 0.049). In addition, there were no differences between the two groups in other indicators. Moreover, significant difference of the serological biomarkers was observed neither in overdominant models of RS4977574 or RS1333045 (details seen in Additional file 2: Table S2).

**Table 3:** Comparisons of serological biomarkers in the dominant model and recessive model

Dominant model of RS4977574	AA	AG + GG	Statistical parameter	*P* value
CKMB (U/L)	16 (12.1–21)	17 (13–22)	− 1.925	0.054
TC (mmol/L)	4.64 (3.75–5.61)	4.62 (3.92–5.43)	− 0.267	0.789
TG (mmol/L)	1.68 (1.13–2.43)	1.82 (1.25–2.49)	− 1.761	0.078
HDL (mmol/L)	1.41 (1.15–1.67)	1.36 (1.12–1.62)	−1.059	0.290
LDL (mmol/L)	2.71 (2.14–3.53)	2.79 (2.22–3.42)	−0.658	0.510
ApoA (g/L)	1.30 (1.16–1.46)	1.29 (1.14–1.46)	−1.038	0.299
ApoB (g/L)	0.91 ± 0.24	0.92 ± 0.22	0.547	0.585
Glu (mmol/L)	5.09 (4.59–6.07)	5.07 (4.62–5.90)	− 0.075	0.940
Bun (mmol/L)	4.94 (3.96–5.80)	4.87 (4.06–5.88)	− 0.150	0.881
Cre (μmol/L)	70 (62–79.75)	71 (62–82)	−0.547	0.584
Recessive model of RS4977574	GG	AA+AG	Statistical parameter	*P* value
CKMB (U/L)	17 (13–22)	17 (13–21)	−0.281	0.779
TC (mmol/L)	4.61 (3.93–5.48)	4.63 (3.88–5.48)	−0.306	0.760
TG (mmol/L)	1.85 (1.26–2.41)	1.75 (1.20–2.50)	−1.060	0.289
HDL (mmol/L)	1.32 (1.10–1.62)	1.38 (1.15–1.64)	− 1.697	0.090
LDL (mmol/L)	2.83 (2.26–3.45)	2.74 (2.15–3.43)	−1.348	0.187
ApoA (g/L)	1.27 (1.13–1.42)	1.30 (1.16–1.48)	−2.191	0.028
ApoB (g/L)	0.90 ± 0.21	0.92 ± 0.23	−1.168	0.243
Glu (mmol/L)	5.04 (4.55–5.80)	5.07 (4.62–6.03)	−1.072	0.284
Bun (mmol/L)	4.87 (4.02–5.61)	4.89 (4.06–5.94)	−1.349	0.177
Cre (μmol/L)	69 (61–81)	71 (62–81)	−1.469	0.142
Dominant model of RS1333045	CC	TC + TT	Statistical parameter	*P* value
CKMB (U/L)	17 (13–22)	16 (13–21)	−0.628	0.530
TC (mmol/L)	4.62 (3.91–5.51)	4.63 (3.88–5.48)	− 0.372	0.710
TG (mmol/L)	1.90 (1.27–2.47)	1.73 (1.18–2.48)	− 1.807	0.071
HDL (mmol/L)	1.32 (1.11–1.62)	1.38 (1.14–1.64)	−1.469	0.142
LDL (mmol/L)	2.94 (2.27–3.51)	2.73 (2.14–3.40)	−2.147	0.032
ApoA (g/L)	1.27 (1.13–1.43)	1.30 (1.16–1.48)	−1.970	0.049
ApoB (g/L)	0.90 ± 0.21	0.92 ± 0.23	−0.989	0.323
Glu (mmol/L)	5.07 (4.57–5.79)	5.07 (4.62–6.03)	−0.915	0.360
Bun (mmol/L)	4.93 (4.04–5.78)	4.87 (4.06–5.90)	−0.612	0.541
Cre (μmol/L)	69 (61–81)	71 (62–81)	−1.175	0.240
Recessive model of RS1333045	TT	TC + CC	Statistical parameter	*P* value
CKMB (U/L)	16 (12.4–21)	17 (13–21.48)	−1.252	0.210
TC (mmol/L)	4.60 (3.74–5.62)	4.65 (3.92–5.44)	− 0.159	0.873
TG (mmol/L)	1.71 (1.15–2.46)	1.81 (1.24–2.48)	− 1.131	0.258
HDL (mmol/L)	1.38 (1.13–1.67)	1.36 (1.12–1.62)	− 0.641	0.521
LDL (mmol/L)	2.64 (2.09–3.48)	2.79 (2.22–3.43)	−1.220	0.223
ApoA (g/L)	1.29 (1.15–1.46)	1.29 (1.14–1.46)	−0.233	0.816
ApoB (g/L)	0.91 ± 0.25	0.92 ± 0.22	−0.576	0.565
Glu (mmol/L)	5.10 (4.59–6.04)	5.05 (4.62–5.92)	− 0.401	0.688
Bun (mmol/L)	4.85 (3.91–5.74)	4.90 (4.06–5.89)	−0.941	0.347
Cre (μmol/L)	71 (62.8–80)	71 (61.05–81)	−0.026	0.979

The serum level of ApoB was examined by the independent samples T test, and the remaining serological biomarkers were examined by the Mann-Whitney test

### The analysis of the rs4977574 and rs1333045 gene polymorphism with CHD

Fisher’s exact test by plink proved that allele G may be associated with a higher risk of CHD (*P* = 0.003, *OR* = 1.371, 95% *CI* = 1.113–1.689) and allele T was likely to reduce the incidence of coronary events (*P* = 0.035, *OR* = 0.798, 95% *CI* = 0.649–0.982) (Table 4). The chi-square test showed consistent results, allele G of rs4977574 (*P* = 0.003, *OR* = 1.371, 95% *CI* = 1.113–1.689) and allele T of rs1333045 (*P* = 0.033, *OR* = 0.798, 95% *CI* = 0.649–0.982) (Table 5).

**Table 4:** Fisher’s exact test of alleles

	A1	A2	*OR* (95% *CI*)	*SE*	*P*
Rs4977574	G	A(reference)	1.371 (1.113–1.689)	0.1063	0.003
Rs1333045	T	C(reference)	0.798 (0.649–0.982)	0.1057	0.035

*OR* Odds ratio, 95% *CI* 95% confidence interval, *SE* Standard error

**Table 5:** The chi-square test of alleles

	A1	A2	*OR* (95% *CI*)	CHISQ	*SE*	*P*
Rs4977574	G	A(reference)	1.371 (1.113–1.689)	8.851	0.1063	0.003
Rs1333045	T	C(reference)	0.798 (0.649–0.982)	4.545	0.1057	0.033

*OR* Odds ratio, 95% *CI* 95% confidence interval, *SE* Standard error

The genotypic distribution and allele frequencies of rs4977574 and rs1333045 in CHDs and controls are shown in Table 6. The rs4977574-A allele frequencies were 0.498 in CHDs and 0.576 in controls, and rs4977574-G allele frequencies were 0.502 in CHDs and 0.424 in controls. The rs1333049-C allele frequency was 0.525 in the CHD group and 0.469 in the control group, and rs1333045-T allele frequencies were 0.475 in CHDs and 0.531 in controls.

**Table 6:** Logistic analysis of the association between SNPs rs4977574 and rs1333045 and CHD risk

Rs4977574 (A > G)	CHD (*n* = 598)	Control (*n* = 257)	*OR*(95% *CI*)	*P* value	AOR*(95% *CI*)	P* value
AA	149	87	1.000 (reference)	0.012	1.000 (reference)	0.005
AG	297	122	1.849 (1.217–2.810)	0.004	2.035 (1.323–3.132)	0.001
GG	152	48	1.421 (1.014–1.993)	0.042	1.452 (1.026–2.054)	0.035
Dominant model (AAvsAG+GG)	149/449	87/170	1.542 (1.122–2.119)	0.008	1.612(1.163–2.235)	0.004
Recessive model (GGvsAA+AG)	152/446	48/209	0.674 (0.468–0.970)	0.033	0.620 (0.427–0.902)	0.012
Allele A frequency	595 (49.75%)	296 (57.59%)	*OR*(95% *CI*)	*P* value	AOR*(95% *CI*)	P* value
Allele G frequency	601 (50.25%)	218 (42.41%)				
HWE	0.871	0.651				
RS1333045 (C > T)	CHD (n = 598)	Control (n = 257)				
CC	168	57	1.000 (reference)	0.108	1.000 (reference)	0.060
TC	292	127	0.641 (0.424–0.970)	0.035	0.599 (0.392–0.915)	0.018
TT	138	73	0.780 (0.541–1.124)	0.183	0.759 (0.522–1.105)	0.151
Dominant model (CCvsTC+TT)	168/430	57/200	0.729 (0.517–1.029)	0.072	0.700 (0.492–0.997)	0.048
Recessive model (TTvsCC+TC)	138/460	73/184	1.322 (0.950–1.842)	0.098	1.392 (0.991–1.955)	0.057
Allele C frequency	628 (52.51%)	241 (46.89%)				
Allele T frequency	568 (47.49%)	273 (53.11%)				
HWE	0.609	0.900				

In the SNP rs4977574, GG represents the homozygote of minor alleles, AG represents the heterozygote, and AA represents the homozygote of major alleles. In the SNP rs1333045, TT represents the homozygote of minor alleles, TC represents the heterozygote, and CC represents the homozygote of major alleles

*AOR* Adjusted odds ratio

*Adjusted for age and gender

The association between the genotypes of SNP rs4977574 and CHD risk was investigated by logistic regression analysis. For rs4977574, the CHD group included 149 individuals with the AA allele, 297 individuals with the AG allele, and 152 individuals with the GG allele. In the control group, 87, 122, and 48 subjects had AA, AG, and GG alleles, respectively. The study results revealed a significant difference between the CHDs and controls in the rs4977574 polymorphism (*P* = 0.012). The frequency of genotype GG (*P* = 0.042, *OR* = 1.421, 95% *CI* = 1.014–1.993) and AG (*P* = 0.004, *OR* = 1.849, 95% *CI* = 1.217–2.810) were increased in the CHD group compared with the control group. In comparison to genotype CC, genotype TC significantly reduced the incidence of CHD (*P* = 0.035, *OR* = 0.641 and 95% *CI* = 0.424–0.970). As reported in Table 6, the subjects with AG + GG (*P* = 0.008, *OR* = 1.542, 95% *CI* = 1.122–2.119) genotypes had a higher risk of CHD than those with AA genotype. Corresponding to this, in a recessive model (GG vs. (AA+AG)), rs4977574 may decrease the incidence of CHD (*P* = 0.033, *OR* = 0.674 and 95% *CI* = 0.468–0.970). Moreover, rs1333045 was more likely to be a protective variant of CHD under the dominant model (CC vs. TC + TT, *P* = 0.048, *AOR* = 0.700, 95% *CI* = 0.492–0.997) when the *P* value was adjusted for age and gender. The gene polymorphisms of two sites in the CHD group and control group were in Hardy-Weinberg equilibrium.

### The relationship between the clinical characteristics and CHD

For further study, we used the receiver operating characteristic (ROC) to describe the clinical indicators associated with CHD. The five indicators of significance are age, CKMB, HDL, Glu and Cre. The area under curve (AUC) is 0.583 for age (*P*<0.001, 95% *CI* = 0.541–0.624); 0.560 for CKMB (*P* = 0.005, 95% *CI* = 0.520–0.601); 0.552 for HDL (*P* = 0.015, 95% *CI* = 0.511–0.593), 0.617 for Glu (*P*<0.001, 95% *CI* = 0.578–0.657); and 0.563 for Cre (*P* = 0.003, 95% *CI* = 0.521–0.605) (as shown in Table 7).

**Table 7:** Receiver operating characteristic curve analyses for predicting CHD prevalence

Variables	AUC (95% *CI*)	*P* value	Cut-off	Sensitivity	Specificity	Youden index
Age (years)	0.583 (0.541–0.624)	< 0.001	59.5	0.562	0.556	0.118
CKMB (U/L)	0.560 (0.520–0.601)	0.005	25.9	0.199	0.938	0.137
TC (mmol/L)	0.518 (0.477–0.559)	0.403	–	–	–	–
TG (mmol/L)	0.525 (0.482–0.567)	0.255	–	–	–	–
HDL (mmol/L)	0.552 (0.511–0.593)	0.015	1.325	0.503	0.385	0.111
LDL (mmol/L)	0.529 (0.487–0.570)	0.185	–	–	–	–
ApoA (g/L)	0.538 (0.500–0.583)	0.081	–	–	–	–
ApoB (g/L)	0.523 (0.482–0.564)	0.280	–	–	–	–
Glu (mmol/L)	0.617 (0.578–0.657)	< 0.001	5.445	0.418	0.794	0.212
Bun (mmol/L)	0.505 (0.463–0.547)	0.809	–	–	–	–
Cre (μmol/L)	0.563 (0.521–0.605)	0.003	73.2	0.463	0.654	0.117

*AUC* Area under curve, *AUC* The closer it is to 0.5, the less predictive it is

### The CHD prevalence and gene-environment interactions on the risk of CHD

In the logistic regression of interactions between CHD, genotypes and variables, crossover analysis was used to reveal the relationship between CHD prevalence and gene-environment interactions on the risk of CHD (details seen in Additional file 1: Table S1).

In the dominant model of rs4977574, the AG + GG genotype showed an increased prevalence of CHD in participants who were ≥ 59.5 years of age (*P* < 0.001, *OR* = 2.304, 95% *CI* = 1.448–3.666) compared to the reference group who had AA genotype who were < 59.5. Females had a lower risk of CHD than males (AA, *P* = 0.004, *OR* = 0.415, 95% *CI* = 0.229–0.753). In addition, the AA genotype without smoking or drinking of the reference group, the AA genotype with smoking (*P* = 0.029, *OR* = 1.831, 95% *CI* = 1.063–3.154), the AG + GG genotype with smoking (*P* < 0.001, *OR* = 2.249, 95% *CI* = 1.448–3.493), drinking (*P* = 0.034, *OR* = 1.803, 95% *CI* = 1.044–3.113) or without smoking (*P* = 0.005, *OR* = 1.824, 95% *CI* = 1.201–2.77), and without drinking (*P* = 0.004, *OR* = 1.661, 95% *CI* = 1.178–2.341) were more likely to have CHD. Patients who do not have hypertension (AG + GG, *P* = 0.004, *OR* = 1.914, 95% *CI* = 1.230–2.977), and who have hypertension (AA, *P* = 0.010, *OR* = 2.028, 95% *CI* = 1.184–3.473; AG + GG, *P* < 0.001, *OR* = 2.487, 95% *CI* = 1.591–3.887) have an increase in CHD prevalence. For the subjects had CKMB< 25.9 (AG + GG, *P* = 0.016, *OR* = 1.515, 95% *CI* = 1.080–2.123), CKMB≥25.9 (AA, *P* = 0.012, *OR* = 3.262, 95% *CI* = 1.296–8.212; AG + GG, *P* < 0.001, *OR* = 6.075, 95% *CI* = 2.982–12.375) existed as a risk factor compared with the AA genotype where CKMB< 25.9. Similarly, the AG + GG genotype with Glu < 5.445 (*P* = 0.004, *OR* = 1.750, 95% *CI* = 1.194–2.564) and the AA or AG + GG genotype with Glu ≥ 5.445 (AA, *P* < 0.001, *OR* = 3.551, 95% *CI* = 1.910–6.603; AG + GG, *P* < 0.001, *OR* = 4.399, 95% *CI* = 2.721–7.113) may have a greater chance of having CHD compared to the reference group (Glu < 5.445, AA). Moreover, the AG + GG genotype increased the risk of CHD in subjects with Cre ≥ 73.2 (*P* < 0.001, *OR* = 2.453, 95% *CI* = 1.565–3.844).

In the recessive model of rs4977574, females had a lower risk of CHD (AG + AA, *P* < 0.001, *OR* = 0.333, 95% *CI* = 0.201–0.551). No drinking also contributed to health (AG + AA, *P* = 0.019, *OR* = 0.625, 95% *CI* = 0.422–0.925). The AG + AA genotype exists as a positive factor for health in subjects with CKMB< 25.9 (AG + AA, *P* = 0.031, *OR* = 0.654, 95% *CI* = 0.444–0.962), Glu < 5.445 (AG + AA, *P* = 0.003, *OR* = 0.521, 95% *CI* = 0.338–0.802), and HDL > 1.325 (AG + AA, *P* = 0.001, *OR* = 0.405, 95% *CI* = 0.236–0.697; GG, *P* = 0.05, *OR* = 0.516, 95% *CI* = 0.267–1.000). However, CKMB≥25.9 (AG + AA, *P* = 0.005, *OR* = 2.675, 95% *CI* = 1.336–5.356), Glu ≥ 5.445 (AG + AA, *P* = 0.034, *OR* = 1.741, 95% *CI* = 1.041–2.911) or Cre ≥ 73.2 (GG, *P* = 0.038, *OR* = 2.122, 95% *CI* = 1.041–4.328) increased the prevalence of CHD.

For rs1333045, in the dominant model, female (CC, *P* = 0.037, *OR* = 0.501, 95% *CI* = 0.262–0.958; TC + TT, *P* < 0.001, *OR* = 0.365, 95% *CI* = 0.226–0.590) or no drinking (TC + TT, *P* = 0.042, *OR* = 0.680, 95% *CI* = 0.469–0.985) possibly reduced the risk of CHD. There are other protective factors, TC + TT genotype with HDL < 1.325 (CC, *P* = 0.023, *OR* = 0.530, 95% *CI* = 0.307–0.916), HDL ≥ 1.325 (CC, *P* = 0.005, *OR* = 0.405, 95% *CI* = 0.217–0.757; TC + TT, *P* = 0.001, *OR* = 0.394, 95% *CI* = 0.232–0.670) or Glu < 5.445 (TC + TT, *P* = 0.006, *OR* = 0.559, 95% *CI* = 0.371–0.843). In contrast, all other interactions increased the risk of CHD. Subjects with age ≥ 59.5 (CC, *P* = 0.006, *OR* = 2.400, 95% *CI* = 1.292–4.459), CKMB≥25.9 (CC, *P* = 0.040, *OR* = 3.118, 95% *CI* = 1.052–9.243; TC + TT, *P* = 0.003, *OR* = 2.825, 95% *CI* = 1.429–5.588), Glu ≥ 5.445 (TC + TT, *P* = 0.009, *OR* = 1.949, 95% *CI* = 1.179–3.225) and Cre ≥ 73.2 (CC, *P* = 0.017, *OR* = 2.221, 95% *CI* = 1.157–4.265) may be associated with CHD.

In the recessive model of rs1333045, compared to the reference group, the likelihood of onset is reduced in females (TT, *P* = 0.003, *OR* = 0.377, 95% *CI* = 0.198–0.717). However, when other factors, such as age ≥ 59.5 (TC + CC, *P* = 0.003, *OR* = 2.042, 95% *CI* = 1.268–3.291), smoking (TT, *P* = 0.029, *OR* = 1.916, 95% *CI* = 1.068–3.435; TC + CC, *P* = 0.003, *OR* = 1.983, 95% *CI* = 1.256–3.130), hypertension (TC + CC, *P* = 0.002, *OR* = 2.048, 95% *CI* = 1.291–3.247), CKMB≥25.9 (TT, *P* = 0.017, *OR* = 3.102, 95% *CI* = 1.226–7.846; TC + CC, *P* < 0.001, *OR* = 5.521, 95% *CI* = 2.684–11.357), Glu ≥ 5.445 (TT, *P* < 0.001, *OR* = 3.550, 95% *CI* = 1.785–7.061; TC + CC, *P* < 0.001, *OR* = 3.654, 95% *CI* = 2.263–5.900), Cre ≥ 73.2 (TT, *P* = 0.047, *OR* = 1.812, 95% *CI* = 1.007–3.260; TC + CC, *P* < 0.001, *OR* = 2.218, 95% *CI* = 1.399–3.517) exist, the risk of CHD increased.

### The synergistic effect between dominant model, recessive model and classical risk factors

The analysis of the associations between gene and classical risk factors is shown in Table 8. We found a positive correlation between the dominant model of rs4977574 and age (*S* = 3.34, *AP* = 0.40, and AP* = 0.70), and the proportion of CHD attributable to the interaction between the dominant model, and age was as high as 70%. The same positive correlation was found between the dominant model and CKMB (*S* = 1.83, *AP* = 0.38, and AP* = 0.45). There is a positive interaction between Glu (*S* = 1.03, *AP* = 0.02, and AP* = 0.03), Cre (*S* = 1.91, *AP* = 0.28, and AP* = 0.48) and the dominant model of rs4977574. In the recessive model of rs4977574 and rs1333045, CKMB shows a positive interaction between them (rs4977574, *S* = 1.23, *AP* = 0.12, and AP* = 0.19; rs1333045, *S* = 1.86, *AP* = 0.38, and AP* = 0.46). In contrast, a negative interaction was found between HDL and the recessive model of rs4977574 (*S* = 0.67, *AP* = 0.73, and AP* = − 0.50) and between HDL and the dominant model of rs1333045 (*S* = 0.57, *AP* = 1.16, and AP* = − 0.76).

**Table 8:** The indexes of the synergistic effect between the dominant model, recessive model and risk factors

	*S*	*AP*	*AP*^*a*^	*RERI*
Variables- Dominant model of rs4977574				
Age- AA/AG + GG	3.34	0.40	0.70	0.913
Gender- AA/AG + GG	1.63	−0.11	0.39	− 0.085
Smoke- AA/AG + GG	0.75	−0.18	−0.33	− 0.406
Drink- AA/AG + GG	0.56	−0.35	−0.80	− 0.639
Hypertension- AA/AG + GG	0.77	−0.18	−0.31	− 0.455
CKMB- AA/AG + GG	1.83	0.38	0.45	2.298
HDL- AA/AG + GG	0.02	−0.30	−48.24	− 0.305
Glu- AA/AG + GG	1.03	0.02	0.03	0.098
Cre- AA/AG + GG	1.91	0.28	0.48	0.691
Variables- Recessive model of rs4977574				
Age- GG/AG + AA	0.25	−0.25	−2.96	−0.273
Gender- GG/AG + AA	0.92	−0.18	−0.09	0.060
Smoke- GG/AG + AA	0.00	−0.62	− 830.35	− 0.618
Drink- GG/AG + AA	0.22	0.47	−3.45	0.417
Hypertension- GG/AG + AA	−0.00	− 0.19	637.34	− 0.186
CKMB- GG/AG + AA	1.23	0.12	0.19	0.316
HDL- GG/AG + AA	0.67	0.73	−0.50	0.297
Glu- GG/AG + AA	−19.62	0.45	1.05	0.779
Cre- GG/AG + AA	0.16	−0.63	−5.19	−0.715
Variables- Dominant model of rs1333045				
Age- CC/TC + TT	0.24	−0.76	−3.18	−1.007
Gender- CC/TC + TT	0.82	0.38	−0.22	0.140
Smoke- CC/TC + TT	0.11	−0.49	−7.75	− 0.524
Drink- CC/TC + TT	0.09	0.42	−10.57	0.406
Hypertension- CC/TC + TT	0.24	−0.37	−3.17	− 0.414
CKMB- CC/TC + TT	1.00	−0.00	− 0.00	− 0.003
HDL- CC/TC + TT	0.57	1.16	−0.76	0.459
Glu- CC/TC + TT	−54.49	0.50	1.02	0.967
Cre- CC/TC + TT	0.23	−0.65	−3.34	− 0.808
Variables- Recessive model of rs1333045				
Age- TT/TC + CC	1.85	0.23	0.46	0.479
Gender- TT/TC + CC	0.82	0.11	−0.22	0.075
Smoke- TT/TC + CC	0.65	−0.27	−0.54	− 0.529
Drink- TT/TC + CC	0.30	−0.81	−2.33	−1.230
Hypertension- TT/TC + CC	0.85	−0.09	−0.17	− 0.180
CKMB- TT/TC + CC	1.86	0.38	0.46	2.097
HDL- TT/TC + CC	−2.37	−0.23	1.42	−0.199
Glu- TT/TC + CC	0.89	−0.09	−0.13	− 0.339
Cre- TT/TC + CC	1.00	−0.00	−0.00	− 0.002

*S* The synergy index S, *AP* Attributable proportion of interaction, *AP*^*a*^ Attributable proportion of pure interaction, *RERI* Relative excess risk of interaction

### Haplotype analysis between rs4977574 and rs1333045

**Fig. 2:**
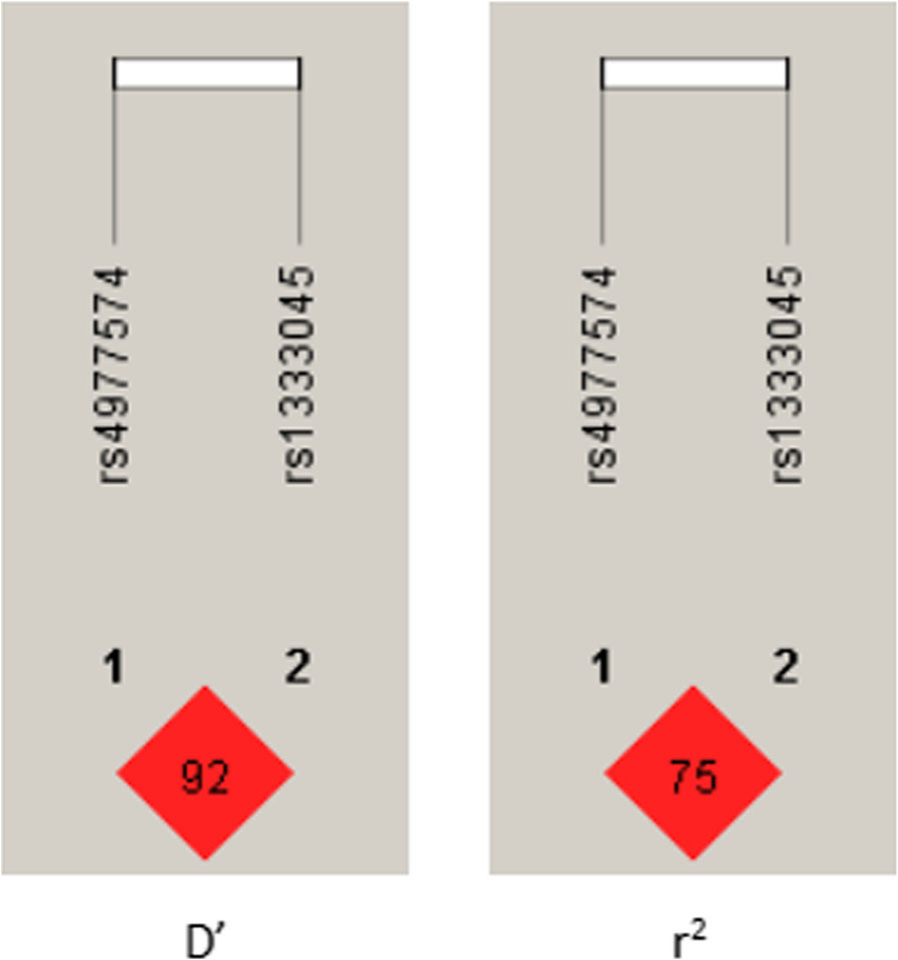
Haplotype analysis between the two groups of rs4977574 and rs1333045

The LD plot on the left indicates the linkage of the gene SNP. *R*^*2*^ refers to the statistical correlation between the two sites. An LD plot was constructed based on the pairwise correlation between the two sites on the 9p21 locus, but unfortunately, it shows that there is no linkage disequilibrium between two sites (*D’* = 0.92 and *r*^*2*^ = 0.75) (Fig. 2).

## Discussion

CHD is a major cause of morbidity and mortality worldwide and represents a tremendous social and economic burden on society [28]. Recent genome-wide association studies have indicated that there is an association between increased susceptibility to coronary artery disease and specific SNPs within the genome that play a role in conjunction with other known traditional CHD risk factors, but the exact mechanism is unclear. Clinical observation has found that atherosclerosis is one of the major pathophysiological mechanisms of CHD [29]. Endothelial cells are monolayer continuous cells covering the inner surface of vessels with significant biological functions, including the regulation of thrombosis and coagulation, dilation of vascular smooth muscle, suppression of platelet adhesion and aggregation [30, 31]. The 9p21.3 risk allele in CHD is associated with altered expression of the CDKN2B-AS1 gene in blood. Evidence has demonstrated that CDKN2B-AS1 is a new susceptibility gene for CHD [32–34]. Both rs4977574 and rs1333045 are ANRILs of the CDKN2B-AS1 gene. Long noncoding RNAs have been shown to have a regulatory role in increasing cell proliferation and decreasing apoptosis in addition to participating in the inflammatory response.

In the present study, we recruited 855 inpatients, 598 cases with CHD confirmed by angiography as the CHD group and 257 subjects with normal coronary artery as the control group. In associating the two groups with SNPs, we analyzed their basic demographic information, including age, gender, smoking status, drinking status, and hypertension, and the following variable values, such as CKMB, TC, TG, HDL, LDL, ApoA, ApoB, Glu, Bun and Cre, which may be related to CHD.

The results of allelic and genotype logic regression showed that the G allele may be associated with a higher risk of CHD (*P* = 0.003, *OR* = 1.371, 95% *CI* = 1.113–1.689), and allele T was likely to reduce the occurrence of coronary events (*P* = 0.035, *OR* = 0.798, 95% *CI* = 0.649–0.982). The frequencies of genotypes GG (*P* = 0.042, *OR* = 1.421, 95% *CI* = 1.014–1.993) and AG (*P* = 0.004, *OR* = 1.849, 95% *CI* = 1.217–2.810) were increased in the CHD group compared with the control group. In comparison to genotype CC, genotype TC significantly reduced the incidence of CHD (*P* = 0.035, *OR* = 0.641 and 95% *CI* = 0.424–0.970). We also evaluated the association between the severity of coronary atherosclerosis and SNP polymorphisms. We found that the degree of vascular stenosis estimated by the Gensini score differed in relation to the rs4977574 genotype. We found that a significantly higher Gensini score appeared in the heterozygous AG genotype and the homozygous GG genotype compared with the AA genotype. This proves that allele G is positively correlated with the severity of CHD. In addition, we analyzed the distribution of clinical parameters in different models. In the recessive model of rs4977574, the genotype AA+AG carriers had significantly higher levels of ApoA (*P* = 0.028) than the GG genotype. In the dominant model of rs1333045, the serum levels of LDL in the CC genotype were higher than in the TC + TT genotype (*P* = 0.032), and the serum levels of ApoA in the CC genotype were significantly lower than in the TC + TT genotype (*P* = 0.049). The conclusion that allele A and allele T are protective factors of CHD should be confirmed more powerfully. Therefore, we suspected that this phenomenon was related to genes affecting the development of CHD by regulating lipid metabolism. Furthermore, we conducted an analysis of CHD prevalence and gene-environment interactions with the occurrence of CHD. It can be seen that genotype GG and AG are more susceptible to CHD than genotype AA when other variables are present. In comparison to genotype CC, genotype TC significantly reduced the incidence of CHD. When traditional risk factors and susceptibility genes coexist, the incidence of CHD is greatly increased.

Nevertheless, the other downside is that in haplotype analysis, it shows that there is no linkage disequilibrium between two sites, but the data showed a degree of association between the two sites that on the same gene. The deeper connection between the two sites needs to be explored further.

In the present study, due to the limited collection of clinical variables, further mechanisms exploring variables were not evaluated in the model building process. At the same time, the sample size of the case-control study was comparatively small, and the age and gender were not well matched. Moreover, the ratio of case and control was not 1:1 due to the consecutiveness of the recruited subjects and the invasiveness of coronary angiography. Therefore, we could not exclude a chance of random positive finding of the genotype association. Further studies utilizing a well-matched group with a larger sample size are warranted to increase the confidence of our findings. However, we can draw some conclusions from this study, and it also lays the foundation for further research in the future.

## Conclusions

We identified that the SNPs tested in the case-control study were significantly associated with CHD. We proved that allele G of rs4977574 may be associated with a higher risk of CHD, and allele T of rs1333045 was likely to reduce the incidence of coronary events. When susceptibility factors (age ≥ 59.5, male, smoking, hypertension, CKMB≥25.9, Glu ≥ 5.445, Cre ≥ 73.2) and susceptibility genes (allele G or allele C) coexist, the incidence of CHD is greatly increased. However, further studies are needed to explain SNPs rs4977574 and rs1333045 in the specific pathogenesis of CHD.

## Supplementary information

**Additional file 1:** **Table S1.** CHD incidence by interactions with environmental factors.

**Additional file 2: Table S2.** Serological biomarkers in the overdominant model.

## Data Availability

The datasets used or analysed during the current study are available from the corresponding author upon reasonable request.

## Abbreviations

ANIRL: Antisense noncoding RNA in the INK4 locus
ApoA: Apolipoprotein A
ApoB: Apolipoprotein B
AUC: Area under curve
Bun: Blood urea nitrogen
CHD: Coronary heart disease
CI: Confidence intervals
CKMB: Creatinephosphokinase isoenzyme
Cre: Creatinine
Glu: Glucose
GWAS: Genome-wide association studies
HDL-C: High-density lipoprotein cholesterol
HWE: Hardy-Weinberg equilibrium
LD: Linkage disequilibrium
LDL-C: Low-density lipoprotein cholesterol
LncRNA: Long noncoding RNA
OR: Odds ratio
PCR: Polymerase chain reaction
RERI: Relative excess risk of interaction
ROC: Receiver operating characteristic
SAP: Shrimp alkaline phosphatase
SE: Standard error
SNPs: Single nucleotide polymorphisms
TC: Total cholesterol
TG: Triglyceride
