# Supplementary material for: Analysis on the polymorphisms of site RS4977574, and RS1333045 in region 9p21 and the susceptibility of coronary heart disease in Chinese population
Source: BMC Med Genet. 2020 Feb 17;21:36. doi: 10.1186/s12881-020-0965-x (PMC7026955; doi:10.1186/s12881-020-0965-x)
Supplement: Supplementary file 1 — Additional file 1: Table S1. CHD incidence by interactions with environmental factors. [file 12881_2020_965_MOESM1_ESM.docx]

**Table S1** CHD incidence by interactions with environmental factors

| Characteristics | Dominant model of rs4977574 | |
| --- | --- | --- |
| Age  <59.5  ≥59.5  Gender  Male  Female  Smoke  No  Yes  Drink  No  Yes  Hypertension  No  Yes  CKMB  ＜25.9  ≥25.9  HDL  ＜1.325  ≥1.325  Glu  ＜5.445  ≥5.445  Cre  ＜73.2  ≥73.2 | AA [OR(95% CI)] P value | AG+GG [OR(95% CI)] *P* value |
|  | 1.000(Reference) <0.001  1.164(0.685-1.977) 0.574  1.000(Reference) <0.001  **0.415(0.229-0.753) 0.004**  1.000(Reference) 0.004  **1.831(1.063-3.154) 0.029**  1.000(Reference) 0.023  1.782(0.820-3.873) 0.145  1.000(Reference) 0.001  **2.028(1.184-3.473) 0.010**  1.000(Reference) <0.001  **3.262(1.296-8.212) 0.012**  1.000(Reference) 0.002  0.690(0.402-1.183) 0.177  1.000(Reference) <0.001  **3.551(1.910-6.603) <0.001**  1.000(Reference) 0.001  1.367(0.794-2.351) 0.259 | 1.227(0.781-1.927) 0.375  **2.304(1.448-3.666) <0.001**  1.451(0.987-2.132) 0.058  0.781(0.499-1.225) 0.282  **1.824(1.201-2.771) 0.005**  **2.249(1.448-3.493) <0.001**  **1.661(1.178-2.341) 0.004**  **1.803(1.044-3.113) 0.034**  **1.914(1.230-2.977) 0.004**  **2.487(1.591-3.887) <0.001**  **1.515(1.080-2.123) 0.016**  **6.075(2.982-12.375) <0.001**  1.621(0.987-2.664) 0.056  1.006(0.626-1.618) 0.979  **1.750(1.194-2.564) 0.004**  **4.399(2.721-7.113) <0.001**  1.396(0.928-2.098) 0.109  **2.453(1.565-3.844) <0.001** |

| Characteristics | Recessive model of rs4977574 | |
| --- | --- | --- |
| Age  <59.5  ≥59.5  Gender  Male  Female  Smoke  No  Yes  Drink  No  Yes  Hypertension  No  Yes  CKMB  <25.9  ≥25.9  HDL  <1.325  ≥1.325  Glu  <5.445  ≥5.445  Cre  <73.2  ≥73.2 | GG [OR(95% CI)] P value | AG+AA [OR(95% CI)] *P* value |
|  | 1.000(Reference) 0.002  1.684(0.873-3.247) 0.120  1.000(Reference) <0.001  0.575(0.289-1.145) 0.115  1.000(Reference) 0.023  1.848(0.937-3.645) 0.076  1.000(Reference) 0.082  0.837(0.329-2.131) 0.709  1.000(Reference) 0.009  1.515(0.786-2.920) 0.215  1.000(Reference) <0.001  2.705(0.902-8.116) 0.076  1.000(Reference) 0.005  **0.516(0.267-1.000) 0.050**  1.000(Reference) <0.001  1.441(0.702-2.957) 0.319  1.000(Reference) 0.002  **2.122(1.041-4.328) 0.038** | 0.681(0.415-1.119) 0.129  1.092(0.663-1.799) 0.729  0.697(0.445-1.093) 0.116  **0.333(0.201-0.551) <0.001**  0.771(0.484-1.227) 0.272  1.001(0.618-1.620) 0.998  **0.625(0.422-0.925) 0.019**  0.879(0.502-1.541) 0.653  0.670(0.410-1.097) 0.111  1.000(0.609-1.642) 0.999  **0.654(0.444-0.962) 0.031**  **2.675(1.336-5.356) 0.005**  0.592(0.338-1.037) 0.067  **0.405(0.236-0.697) 0.001**  **0.521(0.338-0.802) 0.003**  **1.741(1.041-2.911) 0.034**  0.731(0.467-1.143) 0.169  1.138(0.709-1.826) 0.593 |

| Characteristics | Dominant model of rs1333045 | |
| --- | --- | --- |
| Age  <59.5  ≥59.5  Gender  Male  Female  Smoke  No  Yes  Drink  No  Yes  Hypertension  No  Yes  CKMB  <25.9  ≥25.9  HDL  <1.325  ≥1.325  Glu  <5.445  ≥5.445  Cre  <73.2  ≥73.2 | CC [OR(95% CI)] P value | TC+TT [OR(95% CI)] *P* value |
|  | 1.000(Reference) 0.003  **2.400(1.292-4.459) 0.006**  1.000(Reference) <0.001  **0.501(0.262-0.958) 0.037**  1.000(Reference) 0.042  1.764(0.947-3.287) 0.074  1.000(Reference) 0.147  0.875(0.364-2.101) 0.765  1.000(Reference) 0.017  1.747(0.949-3.215) 0.073  1.000(Reference) <0.001  **3.118(1.052-9.243) 0.040**  1.000(Reference) 0.004  **0.405(0.217-0.757) 0.005**  1.000(Reference) <0.001  1.424(0.737-2.750) 0.293  1.000(Reference) 0.004  **2.221(1.157-4.265) 0.017** | 0.923(0.579-1.471) 0.736  1.316(0.824-2.102) 0.250  0.725(0.475-1.105) 0.135  **0.365(0.226-0.590) <0.001**  0.828(0.531-1.291) 0.405  1.068(0.671-1.697) 0.782  **0.680(0.469-0.985) 0.042**  0.962(0.554-1.668) 0.889  0.798(0.501-1.272) 0.343  1.131(0.706-1.810) 0.609  0.711(0.494-1.023) 0.066  **2.825(1.429-5.588) 0.003**  **0.530(0.307-0.916) 0.023**  **0.394(0.232-0.670) 0.001**  **0.559(0.371-0.843) 0.006**  **1.949(1.179-3.225) 0.009**  0.829(0.540-1.271) 0.389  1.242(0.787-1.960) 0.352 |

| Characteristics | Recessive model of rs1333045 | |
| --- | --- | --- |
| Age  <59.5  ≥59.5  Gender  Male  Female  Smoke  No  Yes  Drink  No  Yes  Hypertension  No  Yes  CKMB  <25.9  ≥25.9  HDL  <1.325  ≥1.325  Glu  <5.445  ≥5.445  Cre  <73.2  ≥73.2 | TT [OR(95% CI)] P value | TC+CC [OR(95% CI)] *P* value |
|  | 1.000(Reference) 0.005  1.370(0.776-2.421) 0.278  1.000(Reference) <0.001  **0.377(0.198-0.717) 0.003**  1.000(Reference) 0.026  **1.916(1.068-3.435) 0.029**  1.000(Reference) 0.082  2.257(0.976-5.220) 0.057  1.000(Reference) 0.018  1.757(0.989-3.120) 0.054  1.000(Reference) <0.001  **3.102(1.226-7.846) 0.017**  1.000(Reference) 0.010  0.679(0.381-1.210) 0.189  1.000(Reference) <0.001  **3.550(1.785-7.061) <0.001**  1.000(Reference) 0.005  **1.812(1.007-3.260) 0.047** | 1.193(0.746-0.906) 0.462  **2.042(1.268-3.291) 0.003**  1.214(0.813-1.814) 0.343  0.666(0.421-1.055) 0.083  **1.596(1.032-2.467) 0.035**  **1.983(1.256-3.130) 0.003**  **1.501(1.048-2.149) 0.027**  1.528(0.883-2.643) 0.130  1.470(0.932-2.320) 0.097  **2.048(1.291-3.247) 0.002**  1.323(0.928-1.885) 0.121  **5.521(2.684-11.357) <0.001**  1.380(0.824-2.312) 0.221  0.860(0.523-1.412) 0.551  1.442(0.974-2.136) 0.068  **3.654(2.263-5.900) <0.001**  1.408(0.920-0.715) 0.115  **2.218(1.399-3.517) 0.001** |
