# Supplementary material for: Analysis on the polymorphisms of site RS4977574, and RS1333045 in region 9p21 and the susceptibility of coronary heart disease in Chinese population
Source: BMC Med Genet. 2020 Feb 17;21:36. doi: 10.1186/s12881-020-0965-x (PMC7026955; doi:10.1186/s12881-020-0965-x)
Supplement: Supplementary file 2 — Additional file 2: Table S2. Serological biomarkers in the overdominant model. [file 12881_2020_965_MOESM2_ESM.docx]

| Overdominant model of RS4977574 | AA+GG | AG |  | Statistical parameter | *P* value |
| --- | --- | --- | --- | --- | --- |
| CKMB(U/L)  TC(mmol/L)  TG(mmol/L)  HDL(mmol/L)  LDL(mmol/L)  ApoA(g/L)  ApoB(g/L)  Glu(mmol/L)  Bun(mmol/L)  Cre(µmol/L) | 16(13-21.9)  4.63(3.87-5.52)  1.73(1.22-2.41)  1.41(1.36-1.64)  2.77(2.21-3.48)  1.30(1.14-1.44)  0.90±0.23  5.07(4.56-5.92)  4.92(4.00-5.74)  70(61.63-80.00) | 17(13.4-21)  4.63(3.92-5.40)  1.82(1.23-2.55)  1.37(1.14-1.63)  2.77(2.15-3.40)  1.29(1.16-1.49)  0.93±0.22  5.07(4.63-6.00)  4.87(4.06-6.08)  72(62-82) |  | -1.148  -0.498  -0.678  -0.490  -0.553  -0.927  -1.479  -0.975  -0.147  -1.008 | 0.138  0.619  0.498  0.624  0.580  0.354  0.140  0.330  0.883  0.313 |
| Overdominant model of RS1333045 | CC+TT | TC |  | Statistical parameter | *P* value |
| CKMB(U/L)  TC(mmol/L)  TG(mmol/L)  HDL(mmol/L)  LDL(mmol/L)  ApoA(g/L)  ApoB(g/L)  Glu(mmol/L)  Bun(mmol/L)  Cre(µmol/L) | 17(13-22)  4.62(3.84-5.52)  1.79(1.24-2.46)  1.35(1.12-1.64)  2.79(2.19-3.49)  1.28(1.13-1.44)  0.91±0.23  5.08(4.57-5.91)  4.88(3.98-5.76)  70(62-81) | 17(13-21)  4.65(3.93-5.40)  1.74(1.19-2.48)  1.38(1.14-1.63)  2.75(2.18-3.36)  1.30(1.17-1.49)  0.93±0.22  5.05(4.63-6.02)  4.88(4.07-6.14)  71(62-82) |  | -0.527  -0.190  -0.616  -0.741  -0.839  -1.534  -1.369  -1.152  -1.013  -1.351 | 0.598  0.849  0.538  0.359  0.401  0.125  0.171  0.249  0.311  0.177 |

**Table S2** Serological biomarkers in the overdominant model
